# Effects of *Bacillus amyloliquefaciens* FD777 and *Macleaya cordata* Extract on Performance, Immunity, Gastrointestinal System Microbiome, and Profitability in Holstein Calves

**DOI:** 10.3390/ani15030313

**Published:** 2025-01-23

**Authors:** Mehmet Küçükoflaz, Veli Özbek, Berrin Kocaoğlu Güçlü, Savaş Sarıözkan, Can İsmail Zaman, Erol Aydın, Mustafa Makav, Selma Büyükkılıç Beyzi, Sena Yılmaz Öztaş, Merve Ayyıldız Akın

**Affiliations:** 1 Department of Animal Health Economics and Management, Faculty of Veterinary Medicine, Kafkas University, Kars P.O. Box 36000, Türkiye; 2 Department of Internal Medicine, Graduate School of Health Sciences, Erciyes University, Kayseri P.O. Box 38000, Türkiye; 3 Department of Animal Nutrition and Nutritional Diseases, Faculty of Veterinary Medicine, Erciyes University, Kayseri P.O. Box 38000, Türkiye; bguclu@erciyes.edu.tr (B.K.G.);; 4 Department of Animal Health Economics and Management, Faculty of Veterinary Medicine, Erciyes University, Kayseri P.O. Box 38000, Türkiye; ssariozkan@erciyes.edu.tr; 5 Department of Physiology, Faculty of Veterinary Medicine, Kafkas University, Kars P.O. Box 36000, Türkiye; 6 Department of Animal Science, Faculty of Agriculture, University of Erciyes, Kayseri P.O. Box 38000, Türkiye; 7 Department of Biostatistics, Faculty of Veterinary Medicine, Kafkas University, Kars P.O. Box 36000, Türkiye

**Keywords:** *Macleaya cordata*, *Bacillus amyloliquefaciens*, profitability, Holstein calves

## Abstract

This study was performed to determine the effects of dietary supplementation of *Bacillus amyloliquefaciens* FD777 (BA) and *Macleaya cordata* extract (MCE) on the performance, morbidity and mortality rates, body measurements, immunity, rumen parameters, antioxidant parameters, microbiome level, and profitability of calves during the pre-weaning period. In the study, 51 calves were divided into three groups as one control and two treatment groups considering their age (1 day old), gender (nine females and eight males in each group), and birth weight (37.7 ± 0.4 kg). The calves in the control group (CON) were fed milk without supplements whereas the first treatment group (BA) was fed milk containing 10 mL/day/head of *Bacillus amyloliquefaciens* FD777 and the second treatment group (MCE) was fed milk containing 2 g/day/head of MCE. As a result, supplementing BA and MCE to calf milk had no significant effect on body weight (BW), dry matter intake (DMI), feed efficiency (FE), morbidity and mortality rates, rumen pH, IgG, IgA, and IgM values, and gastrointestinal microbiota (*p* > 0.05). On the other hand, it was determined that body weight gain (BWG), body length, body depth, rump width, withers height change, rump height change, rump width change, and serum GSH level increased significantly in the BA group compared to the control group (*p* < 0.05). According to the partial budget analysis, despite the additional cost of supplementing BA to the calf milk, no calf deaths and lower disease were observed in this group, unlike the other groups, resulting in a lowest calf rearing cost and highest profit. In calves receiving MCE, withers height, rump height, body length, rump width, body depth, chest circumference change, withers height change, rump height change, and rump width change values increased significantly compared with the control group (*p* < 0.05). In conclusion, the results obtained not only reveal the positive effects of BA and MCE on calves during the pre-weaning period, but also encourage the necessity of investigating their effects on the long-term performance of animals and farm economies.

## Simple Summary

The pre-weaning period is a critical time as calves are highly susceptible to disease and their health status can affect lifelong production performance. For this reason, various strategies are being developed for healthy calf production, such as the use of probiotics and phytobiotics in calf feeding. In this study, probiotic and plant extract improved calf health, morbidity and mortality rates, and profitability of calf production. These findings support the use of these feed additives in calf diets to reduce disease risks and increase performance, economic return, and farm sustainability.

## 1. Introduction

Calf health is a field of considerable interest in sustainable animal production. During this critical life stage, appropriate feeding and management strategies affect the growth, development, health, and welfare of calves as well as the genetic capacity of herds, along with future production performance. As the immune system of calves is not fully developed during this period, calves are very sensitive to diseases and environmental stress due to farm conditions; thus, their health risks before weaning are quite high [1,2,3]. The morbidity rate due to enteric infections and calf diarrhea, which are commonly seen in newborn calves in the first three weeks of life, can reach up to 21% in individual herds, with a mortality rate of 5% to 14% [4,5,6,7]. Studies have shown that calf diarrhea causes direct and indirect economic losses to producers owing to the costs for treatment and prophylaxis, growth and retarding, and a subsequent decrease in calf sales prices [8]. However, because neonatal calf health affects the performance and fertility of animals and genetic progress in future periods, it can be speculated that economic losses are not limited to individual calf deaths, growth retardation, and/or treatment costs [9,10,11]. The protection of calves from diseases, optimum nutrient digestibility, and growth rate are largely related to the gastrointestinal microbiota. On the other hand, it has been reported that in modern intensive breeding systems, separating calves from their mothers immediately after birth and feeding them artificially (with whole milk or milk replacer) negatively affects the rapid acquisition of microorganisms from the saliva and feces of their mothers and other cows and slows down the formation of gastrointestinal microbiota [12]. Colonization and development of the intestinal microbiome are very important for the growth and health of calves. For these reasons, multidisciplinary strategies to improve gut health by manipulating the microbiome are increasingly being used to minimize the susceptibility of calves to enteric infections and diarrhea, improve animal health, and enhance growth performance [5,13].

In recent years, the most popular strategies include the use of probiotics and plant extracts in calf rearing. In a study [12], it was determined that probiotics increased growth performance and changed rumen parameters (reduced acetate and increased butyrate) and blood parameters (increased IgA, IgM, and total antioxidant capacity and decreased MDA). In a meta-analysis study, it was reported that probiotics did not significantly affect DMI or FE in calves, but improved daily BWG, and high and significant heterogeneity was observed in the results [14,15]. Pre-weaning calves fed *Lactobacillus plantarum* 299v showed increased growth performance, antioxidant and immune capacity, decreased diarrhea incidence, and diversified fecal bacterial population composition [16]. Although numerous studies have investigated the effects of probiotics on the production performance and health of dairy calves, inconsistent and even contradictory results indicate the need for further studies in this field.

On the other hand, plants and their extracts, which have antimicrobial, antioxidant, and anti-inflammatory properties which contain secondary metabolites produced as part of the defense mechanisms against harmful microorganisms, are also shown among the feed additives that have the potential to improve the health, welfare, and productivity of animals. *Macleaya cordata* (MC), also known as the plum poppy or tree celandine, belongs to the Papaveraceae family (poppy family) and is native to China and Japan. Studies have shown that MCE contains numerous types of chemical compounds, such as alkaloids (allocryptopine, protopine, sanguinarine, and chelerythrine), amino acids, coumarin, volatile oil, saponins, steroids, flavonoids, and carbohydrates, and has antioxidative, anti-inflammatory, antimicrobial, insecticidal, and antitumor effects [17,18]. The majority of the existing information on these feed additives is limited to laboratory animals, pigs, and poultry. Studies in ruminants have suggested that MC reduces ammonia concentration in the rumen and increases microbial protein production, post-ruminal protein digestion, and the number of papillae per cm^2^, but its effects on growth rate, immunity, and microbiota composition in the rumen and small intestine have not been investigated sufficiently [19].

In this study, we aimed to investigate the effects of probiotic supplementation with *Bacillus amyloliquefaciens* FD777 (BA) and *Macleaya cordata* extract (MCE) on performance [body weight (BW), body weight gain (BWG), dry matter intake (DMI), feed efficiency (FE)], morbidity and mortality rate, body measurements [withers height (WH), rump height (RH), body length (BL), body depth (BD), chest circumference (CC), rump width (RW)], immunity (IgA, IgM, IgG), rumen parameters (pH, volatile fatty acids), antioxidant parameters (malondialdehyde, glutathione, catalase, total thiol, natural thiol, disulfide), GIS microbiome level, and profitability of rearing calves during the suckling period.

## 2. Materials and Methods

This study was approved by the Erciyes University Animal Experiments Local Ethics Committee (approval date and number: 6 September 2023 and 23/174).

The animal materials consisted of 51 Holstein calves (27 females and 24 males) provided by a private intensive dairy cattle farm. Calves were selected among the calves born to cows in 2nd and 3rd lactation, which received similar care and feeding programs. The calves were housed in individual hutches (100 cm height, 90 cm width, 160 cm length) made of fiberglass with open and closed areas and straw bedding. All calves were given colostrum equal to 8–10% of their BW in two meals as their first meal within the first 30 min after birth. The calves were divided into 3 groups as a control and 2 treatment groups, containing 17 calves in each, considering their age (1 day old), sex (9 females, 8 males), and birth weight (37.7 ± 0.4 kg). Each calf was given an average of 5.8 L/day of whole milk (38 °C) by bottle for the first 30 days and an average of 6.1 L/day of whole milk (38 °C) between 31 and 60 days. The calves in the control group (CON) were fed milk without additives. The calves in the 1st treatment group (BA) were fed milk containing 10 mL/day *Bacillus amyloliquefaciens* FD777 (Novo Biotic^®^ Atomes Bio. Inc. 3485 Ashby, Ville Saint Laurent, QC, H7W 2N9, Canada) per calf and the 2nd treatment group (MCE) was fed milk containing 2 g/day *Macleaya cordata* extract (Sangrovit^®^ CS, Phytobiotics Futterzusatztoffe GmbH, Rosengasse9, 65343 Eltville, Germany) per calf. During the study, all calves received a mixture of pelleted calf starter feed (90%), alfalfa hay (10%), and water ad libitum. The chemical compositions of the calf starter feed, alfalfa hay, and milk used in the study are presented in Table 1.

Crude ash, crude protein, and crude fat analyses of the calf starter feed and alfalfa hay were performed according to the methods specified by the Association of Official Analytical Chemists [20]. Neutral Detergent Fiber (NDF) and Acid Detergent Fiber (ADF) analyses were performed according to the method described by Van Soest et al. [21], and Non-Fiber Carbohydrate (NFC) analysis was performed according to NRC [22]. The nutrient content of the whole milk fed to the calves was determined every three days using a milk quality analyzer (Milkana^®^ Superior Plus, Mayasan Biotech, Istanbul, Türkiye). The somatic cell count of the milk used for calf feeding was 176,500 cells/mL. Calves were weighed with a 100 g precision scale at the beginning (day 1) and at the end (day 60) of the study, and their BWs were recorded. The average daily body weight gain (DBWG) of each calf was calculated from the obtained data. In the beginning and at the end of the study, biometric body measurements (WH, RH, BL, BD, CC, RW) of all calves were made using a tape measure and a measuring stick according to the method described by Khan et al. [23]. All animals were fed calf starter feed and alfalfa hay after weighing these foodstuffs. After 24 h, the remaining feed in front of the calves was weighed to determine the daily amount consumed. Total DMI was calculated as the sum of DM taken from milk and DM taken from solid feed (calf starter feed + alfalfa hay). The FE was calculated by dividing the total intake of DM (milk DM + solid feed DM) by the total BWG of calves. During the study, the health status of the calves and symptoms of diseases (diarrhea, fever, etc.) were monitored to determine morbidity and mortality rates. The fecal status of each calf was observed and scored each morning. Calves with a fecal score of 3 or 4 were diagnosed with diarrhea [24]. Drug treatment was recorded daily. The medications and treatments given to sick calves were recorded and used in cost analysis. At the end of the study, rumen fluid was collected using a flexible rumen content probe (Forvet^®^, Konya, Türkiye) after 3 h of morning feeding. To minimize salivary contamination, the first 50 mL of rumen fluid was discarded and then approximately 50 mL of rumen fluid (both solid and liquid fractions) was collected. The pH of the rumen fluid was determined immediately using an electronic pH meter (Hanna HI 98127^®^, Cluj County, Romania). Rumen fluid was immediately brought to the laboratory in falcon tubes (50 mL volumetric) in ice bags. Volatile fatty acids (VFA) were analyzed according to Erwin et al. [25]. Briefly, 0.8 mL of fermentation broth and 0.2 mL of solution containing 250 g metaphosphoric acid/L were combined. VFA concentration and molar ratios were determined using an automatic gas chromatograph (GC) (Shimadzu Co. Model 2010+ Shimadzu, Kyoto, Japan). The VFA (acetic, propionic, and butyric acid) concentrations were determined in rumen fluid.

At the end of the study, five calves each were randomly selected from the CON and treatment groups. Calf blood samples were collected into 10 mL serum tubes from the jugular vein before morning feeding. The blood samples were centrifuged at 3500 RPM for 15 min at 25 °C, and the sera were separated and collected into 1.5 mL microtubes. All samples were stored at −20 °C until analysis. IgA, IgM, IgG, malondialdehyde, glutathione, catalase, total thiol, natural thiol, and disulfide analyses were performed from the collected serum samples. The GSH and MDA levels were analyzed applying the methods described by Beutler et al. [26] and Yoshioka et al. [27], respectively. Total thiol (TH) and native thiol (NT) were measured spectrophotometrically using the Total Thiol Assay Kit and the Native Thiol Assay Kit (Rel Assay Diagnostics^®^, Mega Tıp, Gaziantep, Türkiye), according to the kit procedure. Disulfide, disulfide/TT*100, disulfide/NT*100, and NT/TT*100 analyses were calculated using the total thiol and native thiol data [28].

Indirect ELISA (Biotek^®^, Winooski, VT, USA) was used to determine immunoglobulin concentration. This process was performed with a commercial kit (Bio K420^®^, Rochefort, Belgium).

### 2.1. DNA Extraction and Sequencing

On the 60th day of the study, 8 fecal samples per group (4 male and 4 female) were collected in sterile sampling bags and immediately placed in a cooler with ice packs until frozen (−20 °C) after sampling.

During processing, fecal samples were thawed, mixed, and coded, and then the samples were filtered using Sterivex-GP (Millipore, Billerica, MA, USA) with a membrane pore size of 0.22 mm. After filtration, the membrane retaining environmental DNA was used directly for DNA extraction. DNA extraction was performed using the Qiagen Blood & Tissue DNA Purification Kit after homogenization of the obtained filtrate in tubes containing beads using a dry ice homogenizer. All extractions were performed in triplicates and then the isolates were combined in the same pool. The 16SV3 gene region of the obtained DNA was amplified for bacteria. An approximately 200 bp long V3 fragment of 16S rRNA was amplified with primers 16SV3F (5′- ACTCCTACGGGAGGCAGCAGT-3′) and 16SV3R (5′- ACCGCGGCTGCTGGCAC-3′) containing Illumina universal adaptors. All amplifications were performed in triplicate and pooled to be used in the library stage. PCR amplifications were performed with GoTaq^®^ Flexi DNA Polymerase (Promega, Madison, WI, USA) in a total volume of 25 µL on a T100 Thermal Cycler (Bio-Rad) according to the protocol of the manufacturer. Amplified DNA fragments were evaluated on 2% agarose gel in 1× TAE and quantified with a ThermoFisher Qubit 4 Fluorometer. The obtained PCR products were combined and purified using AMPure XP beads (Beckman Coulter Inc., Brea, CA, USA). Library preparations were performed using the Illumina Nextera XT Index Kit v2 according to the “16S Metagenomic Sequencing Library Preparation” protocol. After quantitative measurement was performed with the ThermoFisher Qubit 4 Fluorometer, library sizes were checked on the BioAnalyzer 2100 system and sequenced on the Illumina iSeq 100 platform using 2 × 150 PE chemistry. Bioinformatics analysis was completed via Linux/Unix terminal. Quality controls of forward and reverse read sequences with “fastq” format were performed using the FASTQC program (v0.12.1). The rest of the analysis was performed using the “ObiTools software version 1.2.13” package. Sequences with Phred scores above 20 were aligned and merged using “illuminapairedend”. Then, unmerged reads were cleaned (obigrep), both forward and reverse primers were removed (perl), duplicated data were cleaned (obiuniq), and redundant data from each sample header were sequentially removed (obiannotate). Subsequently, sequences with more than 2 repeats and longer than 100 base pairs were recorded (obigrep) and the abbreviation “c10.l100” was used for them. The “. fasta” files obtained from the ObiTools package were blasted via the SILVA and Geneious Prime interface. Solely those results with a match rate above 97% were taken into account for evaluating the SILVA and Blast results.

### 2.2. Economic Analysis

In the economic analysis, cost elements such as labor, electricity, and water were ignored in the total cost calculation because they were assumed to be equal in all three groups. Calf nutrition and treatment (medicine, additional labor, veterinarian, and dead calf cost) fees were taken into account in the cost calculation. The prices of calf starter feed, alfalfa hay, milk, BA, and MCE used in the cost calculation were taken as 0.29 USD/kg, 0.2 USD/kg, 0.43 USD/L, 36.2 USD/L, and 43.5 USD/kg, respectively. In the calculation of total income, market prices were taken into account and the calf price was taken as USD 14.5/kg BW for Holstein female calves and USD 17.4/kg BW for Holstein male calves.

Partial budget analysis was used to determine the effects of BA and MCE use in calf nutrition on profitability. This analysis is used to determine the positive or negative economic effects of any changes made in enterprise or production systems on the activity carried out. In the analysis, “Additional Income Increase” and “Decreased Costs” have a positive effect on the production system, while “Decreased Income” and “Additional Costs” have a negative effect.

Net Income = (Additional income increase ± Reduced costs) − (Decreasing income ± Additional costs)

The following formula is used to determine the profit situation of the dairy cattle farm: Profit (USD) = Total income − Total cost

### 2.3. Statistical Analysis

The IBM SPSS Version 25.0 package program was used for the statistical analysis of the study. The suitability of the variables for normal distribution was evaluated using the Shapiro–Wilk and Kolmogorov–Smirnov tests.

ANOVA was applied to normally distributed data (Initial BW, BWG, DBWG, DMI, FE, RH, BL, CBL, CC, CCC, CWH, CBD, CRW, rumen pH, IgG, IgM, IgA, Acetic Acid, Propionic Acid, Butyric Acid, A/P, intestinal microbiota, antioxidant parameters, and economic data), and the Kruskal–Wallis test was applied to data that are not normally distributed (Final BW, *Clostridia*, *Lachnospiraceae, Prevotellaceae, Enterobacterales*, and *Proteobacteria*). Duncan’s multiple range test was used for pairwise comparisons. The comparison of the mortality and morbidity status of groups was analyzed with the chi-square test. In addition, *p*-value was taken as 0.05 to determine the level of significance in the study.

Since population parameters were unknown and standard deviation was not determined through a pilot study, standard deviation was not considered necessary in the power analysis. In the sample size calculation with G Power 3.1.9.7 software, the effect size (f = 0.95), margin of error (α = 0.05), power of the test (1 − β = 0.80), and the number of groups = 3 were used, resulting in a total of 15 (3 groups × 5 samples).

## 3. Results

The effects of supplementing MCE and BA to calves’ milk on performance parameters are given in Table 2.

Supplementation of BA and MCE to calves’ milk did not affect the final BW, DMI, and FE ratio (*p* > 0.05). On the other hand, BWG and DBWG were significantly higher in the BA group than in the CON group and numerically higher than in the MCE group (*p* < 0.05; Table 2).

The effects of supplementing BA and MCE to calves’ milk on body measurements are given in Table 3.

At the end of the study (60th day), statistically significant (*p* < 0.05) differences were determined between the CON and treatment groups considering some body measurements. In MCE-supplemented calves, WH, RH, BL, RW, BD, CCC, WHC, RHC, and RWC increased significantly, whereas in the BA group BL, BD, RW, WHC, RHC, and RWC increased significantly compared to the CON group (*p* < 0.05; Table 3).

The effects of supplementing BA and MCE to calves’ milk on IgG, IgA and IgM values are given in Table 4.

Although there was no statistically significant difference between the groups regarding IgG, IgA, and IgM values (*p* > 0.05), a numerical increase was detected in the MCE group (Table 4).

The effects of supplementing BA and MCE to calves’ milk on morbidity and mortality rates are given in Table 5.

In the study, no statistical difference was determined between the CON and treatment groups concerning the morbidity and mortality rates of calves (*p* > 0.05). However, morbidity and mortality rates were numerically lower in the BA and MCE groups than in the CON group. No mortality was observed in the BA group, whereas the highest mortality rate (11.8%) and the lowest number of healthy calves (52.9%) were seen in the CON group (Table 5).

The effects of BA and MCE supplementation to calf milk on rumen VFA and pH values are given in Table 6.

There was no statistical significance between the CON and treatment groups in terms of rumen pH, total VFA, individual VFA (acetate, isobutyrate, propionate, isovalerate, valerate, butyrate) molar proportions, and the ratio of A/P (Table 6; *p* > 0.05).

The main bacterial taxa in the feces of the groups are given in Table 7.

Firmicutes ranged from 47% to 74%, Bacteroidia from 23% to 50%, and Proteobacteria from 0.3% to 4% in the fecal microbiota. The supplementation of BA and MCE to calf milk had no significant effect on the intestinal microbiota of calves (Table 7).

**Figure 1:**
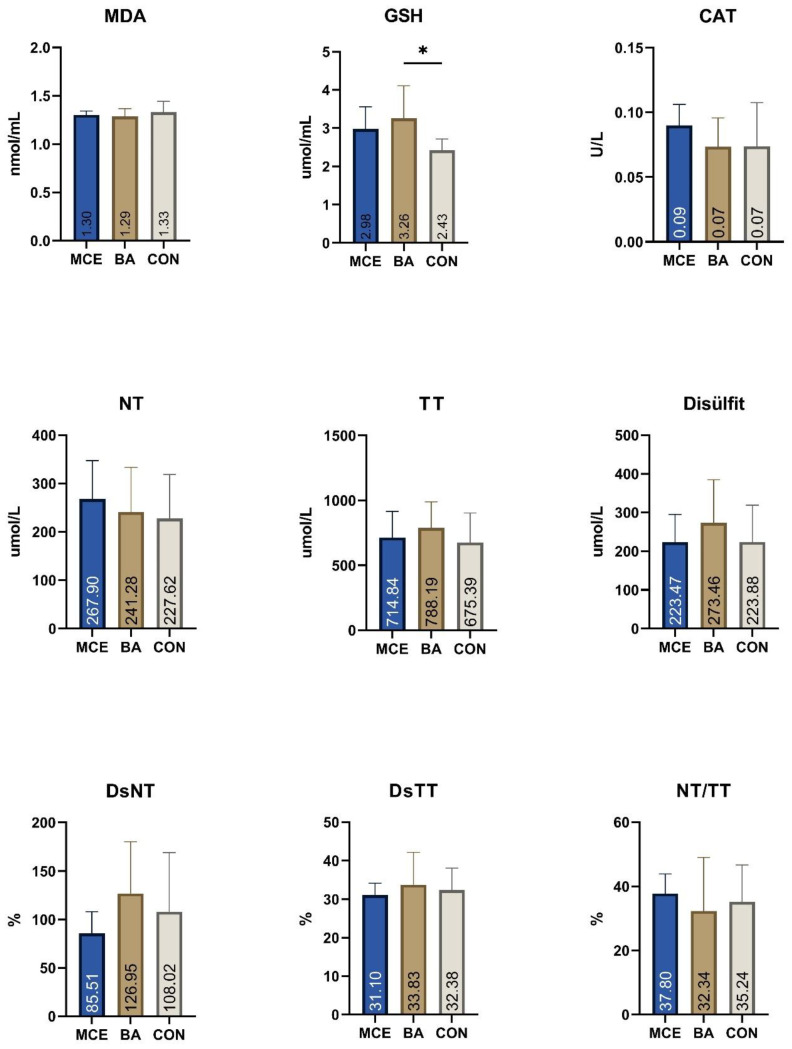
Antioxidant parameters of the study groups. (Glutatyon (GSH), malondialdehyde (MDA), catalase (CAT), native thiol (NT), total thiol (TT), disulfide (Ds), disulfide/native thiol × 100 (DsNT), disulfide/total thiol × 100 (DsTT), native thiol/total thiol × 100 (NT/TT)) * *p* < 0.05.

Considering the GSH parameter, a significant increase was seen in the BA group compared to the CON group. No statistically significant difference was detected in MDA, CAT, NT, TT, disulfide, DsNT, DsTT, and NT/TT data (Figure 1).

The effects of MCE and BA supplementation in calves’ milk on nutrition, treatment, and total costs are given in Table 8.

A statistically significant difference was found between the CON, BA, and MCE groups in terms of nutritional cost (*p* < 0.05). The group with the lowest calf feeding cost was CON, while the group with the highest calf feeding cost was BA. The treatment cost was the lowest in the BA group, whereas it was the highest in the CON group (*p* < 0.05). The difference between the groups in terms of total cost was not statistically significant (*p* > 0.05). The lowest total calf rearing cost was calculated in the BA group as USD 190.9 (Table 8).

The economic impact of BA and MCE supplementation to calf milk is given in Table 9.

In the present study, the total income in the BA group was higher than in the other groups (*p* < 0.05). No statistically significant difference was found between the groups concerning total cost and net profit (*p* > 0.05). However, the MCE and BA groups provided USD 238.2 and USD 444.2 more profit per calf than the CON group, respectively (Table 9).

## 4. Discussion

There is increasing interest in feed additives that support the development of GIS and immunity to optimize the general health status and growth rates of calves before weaning, which is a sensitive period in calves’ development against environmental, metabolic, and pathogenic factors [29,30]. It has been reported that probiotics and phytobiotics are effective in increasing nutrient digestion and absorption and growth performance and in reducing the incidence of enteric diseases by affecting the intestinal microflora and immune capacity. Bacillus species have become more popular in recent years because of their advantages (shelf life, resistance to high temperatures, UV radiation, and low stomach pH) due to the formation of spores as well as their ability to produce various enzymes such as protease, amylase, and lipase [31].

This study showed that in the BA-supplemented group, DMI, and FE values increased numerically and BWG and DBWG increased significantly compared to the CON group. These findings are similar with the results of studies reporting that *Bacillus subtilis* or *Bacillus amyloliquefaciens* supplementation increased calves’ BWG [31,32] and the body measurements [31] of calves, but did not affect FI [33] and FE [34]; however, it differed from the studies reporting increased FE [31,35] and FI [31]. At the end of the study, significant increases in BL, BD, RW, WHC, RHC, and RWC in parallel with the BWG were determined in the BA-supplemented group compared to the CON group. Noori et al. [36] reported that probiotics increased WH in their study on Holstein calves, and they suggested that this increase may be attributed to the effects of probiotics on increasing the bioavailability of essential minerals such as calcium, phosphorus, and magnesium.

MCE has a bitter taste because it contains isoquinoline alkaloids, including sanguinarine, and chelerythrine. However, it has the potential to increase FI since the bitter taste at low levels can mask other variable tastes that may temporarily reduce feed intake [37]. In the present study, the determination of the highest DMI in the MCE group supported this idea. In addition, some literature suggests that MCE increases intestinal barrier function [38], alleviates intestinal inflammatory response [39], regulates intestinal morphology [40], and thus increases DMI by promoting ruminal functions, nutrient digestion, and utilization [19,41]. In this study the higher DMI resulted in significant increases in WH, RH, BL, RW, BD, CCC, WHC, RHC, and RWC at the end of the trial in the MCE-supplemented calves. However, like similar results reporting that MCE did not affect end-of-trial BWG [19,39,42], DBWG [37,42,43,44], and FE [19,42,43] in ruminants, the MCE supplementation did not significantly affect calves’ FE, end-of-trial BW, and DBWG in the present study. It is thought that the different results obtained in studies may be related to the dose of MCE and the study period. Matulka et al. [37] reported that a low dose (2 g/day) of MCE reduced FI in calves, but high doses (5 and 10 g/day) had no effect.

Due to the interplay between the intestinal microbiota, GIS epithelium, and GIS-associated immune cells, as well as the impact of the phytobiotics’ bioactive compounds, probiotics were shown to have an immunological modulatory effect [45]. The use of sanguinarine derived from MCE has been approved as a feed additive in the EU countries and China and has been shown to improve growth performance and stimulate immune activity in farm animals [46,47,48,49]. Serum immunoglobulins including IgA, IgG, and IgM are an important indicator of humoral immunity and are produced by B-lymphocytes to prevent and resist infection.

While it has been reported that IgA levels, which play an important role in mucosal immunity, increased numerically in calves supplemented with BA [31] and significantly in lambs supplemented with MCE [47], no significant change was observed in the BA or MCE groups in our study. IgM is the first antibody produced in response to infection and plays a critical role in the early stages of the immune response. Although no significant increase in IgM levels was observed in the MCE and BA groups in our study, it was found that IgM levels were numerically higher in the BA group compared to the control group. This observation supports the findings of Du et al. [31], who reported that serum IgA, IgM, and IgG levels were increased in calves supplemented with *B. amyloliquefaciens/B. subtilis*, though not significantly. The increase in IgM levels in the BA group of our study was associated with a lower morbidity rate and no mortality compared to the CON and MCE groups, indicating that early immune responses were enhanced in the calves. IgG is the most abundant antibody in serum and plays a critical role in long-term immunity and pathogen neutralization. As with IgM, higher IgG levels were observed in the BA group compared to the control group, supporting the potential of BA supplementation to enhance the immune system, as reported by Du et al. [31]. The increase in IgG levels suggests that BA supplementation may have the potential to enhance long-term immunity in calves. Although MCE did not show a significant effect on immunoglobulin levels in our study, a trend toward an increase in serum immunoglobulin levels was observed. This is in line with the study by Jiao et al. [46], who reported that the concentrations of IgA, IgG, and IgM antibodies were significantly increased in the MCE-supplemented serum of lambs.

The most dominant genera in all groups were *Lachnospiraceae, Oscillospiraceae*, and *Prevotellaceae*, respectively. Similarly, previous studies have shown that Firmicutes and Bacteroidetes are the main microbial groups in the intestinal flora of healthy calves [31,50,51]. From a phylum-level perspective, Firmicutes ranged from 47% to 74%, Bacteroidia from 23% to 50%, and Proteobacteria from 0.3% to 4% in the fecal microbiota of healthy Holstein calves in the neonatal period. At the class level, Clostridia ranged from 42% to 68%, Bacilli ranged from 1% to 8%, and Bacteroidales ranged from 23% to 44%. The early gut microbiota plays a vital role in the long-term health of the host and the diversity, composition, and relative abundance of the gut flora are affected by probiotic or phytobiotic administration [31]. However, in the current study, no significant difference was determined between the groups regarding fecal microbiome composition. Zhang et al. [52] stated that the effect of probiotics on the rumen microbial population was greater than their effect on fecal microbial populations. This may be one of the reasons why BA did not affect the fecal microbiome composition in our study. Similar to the results of studies reporting no significant effect of MCE supplementation on the microbiota composition in calves (13 weeks old) during the suckling period [41,53], the lack of significant change in the fecal microbiota of calves receiving MCE in our study can be explained by the dose used, as stated in the literature, which reported that the antimicrobial properties of plant extracts may be dose-dependent [54].

Rumen function and the stability of the intraruminal milieu could be reflected by fermentation characteristics such as ruminal pH and VFA profiles. In the current study, supplementation of MCE and BA in diets did not affect ruminal pH, which is consistent with the lack of treatment effect on the total VFA concentration. While the results obtained were found to be compatible with some studies [19,35,53], they were found to be different from others [33,50].

The results of the antioxidant indices showed that there was no significant change in the parameters except GSH in calves provided either BA or MCE in milk. GSH is a non-enzymatic antioxidant with a tripeptide structure that can scavenge free radicals, detoxify, and protect the integrity of the erythrocyte membrane and cellular immunity. GSH, which activates the antioxidant mechanism in specific glutathione redox cycles, is an important indicator for measuring the body’s antioxidant capacity. In this study, the determination of a significant increase in GSH level in calves fed BA compared to the control group supports previous studies suggesting that probiotics protect against oxidative stress by increasing antioxidant capacity [55].

In the present study, it was determined that the nutrition cost increased significantly in the BA and MCE groups due to the extra cost of additives, but the total cost of calf rearing did not change. Due to lower disease and mortality, treatment costs decreased significantly in the BA group and numerically in the MCE group. In the MCE and BA groups, USD 171 and USD 319.6 more total income per calf and USD 238.2 and USD 444.2 more profit per calf were calculated compared to the CON group, respectively. Previous studies [56,57] reporting the positive contribution of probiotics to farm economies by reducing mortality and morbidity rates support the findings of the current study. In addition, similarly with this study, the results of the previous studies have shown an increase in profitability with using probiotics in calf rearing [58]. The results obtained in this study that differ from some literature findings support the view that the effectiveness of plant extracts and probiotics is affected by many factors such as differences in the composition of the products used in the studies, application methods, period and dosage, and environmental stress factors, as well as the age and health status of the animals.

## 5. Conclusions

Based on the findings of this study, despite no positive effect for some variables (feed intake/efficiency, immunity, rumen, and most of antioxidant parameters), supplementing BA and MCE as a probiotic and phytobiotic source to calf milk may have potential effects on BWG, body measurements, health status, and calf rearing costs and profit. As determined in the BA- and MCE-supplemented groups, lower morbidity/mortality rates, along with the fast and healthy growth of calves, are the priorities of dairy farms for sustainability and higher profitability. On the other hand, the results obtained not only reveal the positive effects of BA and MCE on calves during the pre-weaning period but also encourage the necessity of investigating their effects with different doses and periods on the long-term performance of animals and farm economies.

## Figures and Tables

**Table 1:** The chemical compositions of calf starter feed, alfalfa hay, and milk.

Nutrient Composition	Calf Starter	Alfalfa Hay	Whole Milk
DM, % (feed basis)	89.57	92.86	12.76
CP, DM%	19.00	16.47	3.39
Fat, DM%	-	-	3.70
Ash, DM%	7.71	11.52	-
EE, DM%	4.08	3.40	-
CF, DM%	7.30	21.10	-
ADF, DM%	9.97	30.52	-
NDF, DM%	23.98	38.58	-

DM—dry matter; CP—crude protein; EE—diethyl ether extract; CF—crude fiber; ADF—acid detergent fiber; NDF—neutral detergent fiber.

**Table 2:** The effects of supplementing MCE and BA to calves’ milk on performance.

Parameters	CON (x ± SEM)	BA (x ± SEM)	MCE (x ± SEM)	*p*-Value
Initial BW, kg	37.36 ± 1.46	38.11 ± 1.16	37.73 ± 0.97	0.909
Final BW, kg	73.18 ± 2.39	78.71 ± 0.76	76.38 ± 0.84	0.102
BWG, kg	35.82 ± 1.53 ^a^	40.61 ± 1.08 ^b^	38.65 ± 1.16 ^ab^	0.037
DBWG, g	511.68 ± 21.86 ^a^	580.10 ± 15.47 ^b^	552.20 ± 16.56 ^ab^	0.037
DMI, g/day	530.25 ± 47.68	571.62 ± 26.27	594.71 ± 25.15	0.405
FE, gDMI/gBWG	1.03 ± 0.08	0.99 ± 0.04	1.08 ± 0.05	0.464

BW—body weight; BWG—body weight gain; DBWG—daily body weight gain; g DMI—dry matter intake; FE—feed efficiency; CON—fed a basal diet; MCE—fed a basal diet supplemented with 2 g/day *Macleaya cordata* extract; BA—fed a basal diet supplemented with 10 mL/day *Bacillus amyloliquefaciens* FD777. a-b-ab: different superscripts with different letters show statistically significant values (*p <* 0.05).

**Table 3:** The effects of supplementing BA and MCE to calves’ milk on body measurements.

Parameters	CON (x ± SEM)	BA(x ± SEM)	MCE (x ± SEM)	*p*-Value
Withers height on day 0, cm	77.82 ± 1.03	77.14 ± 0.74	76.85 ± 0.85	0.814
Withers height on day 60, cm	85.45 ± 0.97 ^a^	87.07 ± 0.49 ^a^	90.54 ± 0.62 ^b^	0.0001
Change in withers height, cm	7.64 ± 0.69 ^a^	9.93 ± 0.55 ^b^	13.39 ± 0.84 ^c^	0.0001
Rump height on day 0, cm	82.82 ± 1.02	81.43 ± 0.64	81.31 ± 0.88	0.401
Rump height on day 60, cm	91.82 ± 1.09 ^a^	93.14 ± 0.67 ^a^	97.77 ± 0.70 ^b^	0.0001
Change in rump height, cm	9.00 ± 0.82 ^a^	11.71 ± 0.60 ^b^	16.46 ± 0.82 ^c^	0.0001
Body length at day 0, cm	72.82 ± 1.01	72.57 ± 0.54	72.38 ± 0.72	0.924
Body length at day 60, cm	77.55 ± 1.34 ^a^	81.07 ± 0.90 ^b^	81.77 ± 1.19 ^b^	0.035
Change in body length, cm	4.73 ± 1.51	8.50 ± 0.98	9.38 ± 1.68	0.069
Body depth at day 0, cm	33.45 ± 0.47	33.71 ± 0.27	33.69 ± 0.44	0.726
Body depth at day 60, cm	33.57 ± 0.79 ^a^	34.07 ± 0.50 ^b^	34.54 ± 0.51 ^b^	0.032
Change in body depth, cm	0.12 ± 0.46	0.36 ± 0.60	0.85 ± 0.72	0.082
Chest circumference at day 0, cm	78.45 ± 1.01	78.64 ± 0.75	78.00 ± 0.74	0.846
Chest circumference at day 60, cm	97.45 ± 1.24	99.64 ± 0.57	99.85 ± 0.69	0.433
Change in chest circumference, cm	19.00 ± 0.66 ^a^	21.00 ± 0.75 ^ab^	21.85 ± 0.85 ^b^	0.048
Rump width on day 0, cm	24.18 ± 0.44	24.14 ± 0.38	24.07 ± 0.29	0.997
Rump width on day 60, cm	27.36 ± 0.53 ^a^	29.57 ± 0.58 ^b^	32.38 ± 0.43 ^c^	0.0001
Change in rump width, cm	3.18 ± 0.40 ^a^	5.43 ± 0.62 ^b^	8.31 ± 0.41 ^c^	0.0001

a-b-c-ab: different superscripts with different letters show statistically significant values (*p* < 0.05).

**Table 4:** Effects of supplementing BA and MCE to calves’ milk on IgG, IgA, and IgM values.

Parameters	CON (x ± SEM)	BA (x ± SEM)	MCE (x ± SEM)	*p*-Value
IgG (mg/dL)	382.50 ± 15.61	406.80 ± 14.13	408.33 ± 20.28	0.482
IgM (mg/dL)	128.00 ± 3.24	131.40 ± 8.89	132.33 ± 7.33	0.918
IgA (mg/dL)	92.50 ± 18.98	90.00 ± 10.61	103.33 ± 27.44	0.870

**Table 5:** Effects of supplementing BA and MCE to calves’ milk on morbidity and mortality rates (%).

Health Status/Groups	CON	BA	MCE
Healthy, %	52.9	88.2	70.6
Morbidity, %	35.3	11.8	23.5
Mortality, %	11.8	0.0	5.9
Statistical values	N = 51. X2 = 5.50. Sd = 4. *p* = 0.240		

**Table 6:** The effects of BA and MCE supplementation to calf milk on pH and molar proportions of VFA in the rumen.

Parameters	CON (x ± SEM)	BA (x ± SEM)	MCE (x ± SEM)	*p*-Value
Rumen pH	6.41 ± 0.14	6.40 ± 0.19	6.38 ± 0.19	0.987
Total VFA, mmol/L	91.97 ± 1.29	89.97 ± 1.14	93.76 ± 1.74	0.184
Acetate, mol/100 mol	63.09 ± 1.38	62.02 ± 0.32	64.30 ± 1.31	0.414
Iso-butyrate, mol/100 mol	0.52 ± 0.05	0.59 ± 0.04	0.69 ± 0.07	0.121
Propionate, mol/100 mol	25.67 ± 1.25	26.37 ± 0.95	23.98 ± 1.05	0.299
Iso-valerate, mol/100 mol	0.52 ± 0.05	0.57 ± 0.03	0.59 ± 0.04	0.437
Valerate, mol/100 mol	1.45 ± 0.06	1.46 ± 0.06	1.42 ± 0.07	0.918
Butyrate, mol/100 mol	8.77 ± 0.43	9.00 ± 0.37	9.02 ± 0.48	0.900
A/P	2.54 ± 0.19	2.39 ± 0.11	2.75 ± 0.18	0.295

**Table 7:** Relative abundance of main bacterial taxa that exhibited differences in feces from MCE- and BA-supplemented calves.

Microbiota	CON (x ± SEM)	BA (x ± SEM)	MCE (x ± SEM)	*p*-Value
Phylum.%				
Firmicutes	59.75 ± 1.44	59.60 ± 2.73	60.00 ± 6.56	0.998
Bacteroidia	37.00 ± 1.58	37.40 ± 2.27	36.33 ± 6.84	0.983
Proteobacteria	0.65 ± 0.15	1.46 ± 0.64	1.97 ± 0.61	0.137
Class.%				
Clostridia	55.00 ± 1.47	53.60 ± 3.41	54.00 ± 6.00	0.913
Bacilli	3.25 ± 0.75	3.80 ± 0.37	5.00 ± 1.15	0.301
Bacteroidales	37.00 ± 1.58	37.40 ± 2.27	34.33 ± 4.84	0.738
Genus.%				
Ruminococcaceae	5.50 ± 0.29	5.40 ± 0.51	6.33 ± 0.88	0.502
Lachnospiraceae	19.50 ± 0.29	21.40 ± 0.81	14.33 ± 6.69	0.226
Oscillospiraceae	14.50 ± 1.93	11.40 ± 1.33	11.33 ± 0.88	0.300
Prevotellaceae	18.25 ± 0.48	18.60 ± 0.68	14.33 ± 2.60	0.280
Enterobacterales	0.27 ± 0.21	0.70 ± 0.37	0.75 ± 0.63	0.686

**Table 8:** The effects of MCE and BA supplementation to calf milk on nutrition, treatment, and total costs (USD).

Cost Items/Group	CON (x ± SEM)	BA (x ± SEM)	MCE (x ± SEM)	*p*-Value
1. Nutrition, USD	163.8 ± 0.8 ^a^	186.3 ± 0.5 ^c^	170.3 ± 0.4 ^b^	<0.001
- Milk, USD	153.9	153.9	153.9	
- Feed, USD	9.9	10.7	11.2	
- Additive, USD (BA/MCE)	0.0	21.7	5.2	
2. Treatment, USD	151.7 ± 84.1 ^b^	4.6 ± 3.5 ^a^	78.0 ± 68.8 ^ab^	0.029
- Drug, USD	18.6	3.4	10.9	
- Veterinary, USD	7.7	1.1	3.6	
- Labor, USD	0.5	0.1	0.3	
- Cost of dead calf, USD	124.9	0.0	63.2	
3. Total cost, USD (3 = 1 + 2)	315.5 ± 84.0	190.9 ± 3.6	248.3 ± 68.7	0.196

a-b-c-ab: different superscripts with different letters show statistically significant values (*p <* 0.05).

**Table 9:** Economic impact of supplementing BA and MCE in calves’ milk.

Parameters/Groups	CON (x ± SEM)	BA (x ± SEM)	MCE (x ± SEM)	*p*-Value
1. Total cost, USD/calf	315.5 ± 84.0	190.9 ± 3.6	248.3 ± 68.7	0.196
2. Total income, USD/calf	952.0 ± 128.4 ^a^	1271.6 ± 35.2 ^b^	1123.0 ± 92.4 ^ab^	0.025
3. Net profit, USD/calf (3 = 2 − 1)	636.5 ± 208.3	1080.7 ± 33.9	874.7 ± 158.7	0.058

a-b-ab: different superscripts with different letters show statistically significant values (*p* < 0.05).

## Data Availability

The data supporting this study’s findings are available from the corresponding author upon reasonable request.
